# Environmental Factors Driving the Spatial Distribution Pattern of Venerable Trees in Sichuan Province, China

**DOI:** 10.3390/plants11243581

**Published:** 2022-12-19

**Authors:** Chunping Xie, Meng Li, C. Y. Jim, Dawei Liu

**Affiliations:** 1 College of Sciences, Qiongtai Normal University, Haikou 571127, China; 2 Co-Innovation Center for the Sustainable Forestry in Southern China, College of Biology and the Environment, Nanjing Forestry University, Nanjing 210037, China; 3 Department of Social Sciences, Education University of Hong Kong, Hong Kong 999077, China; 4 Nanjing Forest Police College, Nanjing 210023, China

**Keywords:** heritage tree, species range, climate change scenario, bioclimatic factor, elevation factor, tree conservation

## Abstract

Venerable trees are important natural resources and cultural heritage, offering historical, ecological, social and economic value. However, global warming and anthropogenic activities have threatened their welfare and survival. A comprehensive understanding of their current and future spatial patterns, vis-á-vis environmental conditions, can inform the co-management of sustainable resource use and conservation. We employed the existing spatial occurrence data and environmental variables (bioclimate and elevation) to simulate the optimal habitats for venerable trees in China’s Sichuan Province. We evaluated the current and future climate scenarios of 2100 with double CO_2_ concentration. The BIOCLIM and QGIS spatial analyses assessed the primary factors of geographical distribution. The results identified 10,720 venerable trees from 123 species, 81 genera and 42 families. *Cupressus funebris* dominated, with the maximum importance value, followed by *Ginkgo biloba*, *Ficus virens* var. *sublanceolata*, and *Phoebe zhennan*. The elevation distribution of tree abundance and species richness demonstrated a unimodal pattern, skewing to the low-elevation end, with a concentration in the 600–1500 m low-medium altitude. The majority of trees and excellent habitats were found in eastern Sichuan with a less harsh terrain and climate. The bio3 (isothermality) and bio7 (temperature annual range) factors significantly influenced tree occurrence. Temperature imposed a greater effect on distribution than moisture under the current climate scenario. For the future climate-change scenario, the suitable habitats were predicted to maintain an overall stable pattern, with largely contiguous expansions of better habitats. However, climate warming would shrink the excellent habitats on the plains. The findings can inform strategies and guidelines for venerable-tree conservation in Sichuan. Furthermore, vulnerable areas could be identified. The future range expansion sites could be enlisted to cultivate new trees to replenish the venerable-tree pool. Habitat patches that remain sustainable could provide refugia with the potential for protected-area designation.

## 1. Introduction

Venerable trees, also known as large old trees or heritage trees [1], denote living chronicles of natural environmental changes and bear witness to the vicissitudes of urban development and other human impacts [2,3]. Venerable trees are crucial elements of natural ecosystems, offering essential ecological services, such as soil and water conservation, environmental pollution reduction, carbon sequestration, microclimate regulation, and animal habitat provision [2,4,5]. They are widely respected and often applauded as “living relics” or “living fossils” in different parts of the world [3,6]. In many regions, venerable trees are closely associated with regional culture, aesthetics, religion, history, mythology and totems, echoing the relished heritage of history and culture [7,8]. However, due to climate change, the increasing frequency of extreme weather events, and intensifying human perturbations, venerable trees have suffered a sharp decline in various locations [9,10], degrading the integrity of local ecosystems and biodiversity [11]. Therefore, it is important to preserve the surviving venerable trees and promote the cultivation of potential ones to sustain acceptable traditional resource utilization and conservation.

Since the 1980s, Chinese forestry departments have studied venerable trees extensively. Some advances have been achieved in building systematic tree databases and research on tree health, disease, physiology, and rejuvenation technology [12,13]. However, the coverage and scope of venerable tree studies in China remain deficient, leaving some knowledge and practice gaps. Thus far, the relevant investigations have focused primarily on the spatial distribution pattern of venerable trees, conducted at the large city or county scale or in specific locations [13,14,15]. The large-scale spatial and temporal distribution characteristics are rarely explored [16,17]. At such a micro-scale, it is not feasible to evaluate climate as an environmental factor in venerable-tree distribution. The study of the large-scale spatial pattern should be conducted at the provincial level or above.

Interactions between organisms and the climate significantly impact species distribution [18]. Climate change can modify an ecosystem’s key ecological processes, habitat conditions, and species composition and interactions, which are often irreversible [19]. In the climate-change context, the influence of bioclimatic factors on the geographic pattern of venerable trees is worthy of in-depth scientific inquiry.

The drivers of the spatial pattern of venerable trees have recently received much research attention [13,14,15,17,20,21]. The species composition, abundance and distribution patterns of venerable trees are often affected by a combination of factors, such as natural environment, local customs, religious beliefs, historical changes and economic development [2,22]. For instance, the distribution of large old *Ginkgo biloba* in China is contingent upon hydrological, thermal and soil conditions, setting limits to its range [23]. The control of environmental factors on the distribution of other venerable-tree species has been shown at a large scale [2,16,17]. Consequently, understanding the key factors circumscribing the distribution of venerable trees from a large spatial scale is conducive to formulating conservation strategies from a macroscopic perspective.

In recent years, the development of statistics and geographic information technology (GIS) have facilitated the development of Species Distribution Models (SDMs) for predicting the spatial distribution of species [24]. The technique has been widely applied in invasion ecology, conservation ecology, and the prevention of the spatial transmission of infectious diseases [25,26,27].

Many SDMs have been developed to assess the relationship between species distribution and selected environmental variables. They include BIOCLIM, DOMAIN, Ecological Niche Factor Analysis (ENFA), Mahalanobis Distance (MD), Maximum Entropy (MaxEnt), GLM (Generalized Linear Model), GAM (Generalized Additive Model), and CART (Classification and Regression Tree) [28,29].

This study examined the distribution patterns of venerable trees in the Sichuan Province of China, under the current and future climate change scenarios, using the BIOCLIM model. The study objectives are to: (1) identify and assess the significant climatic factors influencing the current distribution of venerable trees; (2) predict the suitable habitats for future growth of venerable trees based on their current distribution data; and (3) forecast the changes in suitable habitats for venerable-tree growth under a climate-change scenario. The findings could contribute to conserving venerable trees, selecting suitable ex situ conservation sites, and managing the sustainable utilization of a precious resource.

## 2. Results

### 2.1. Species Composition and Importance Value

There are 10,720 venerable trees composed of 123 species in Sichuan, belonging to 42 families and 81 genera. They were divided into four categories, based on frequency: dominant (>100 individuals per species), common (10–100 individuals per species), rare (2–9 individuals per species), and solitary (one individual per species) (Table S3). *Cupressus funebris* achieved overwhelming supremacy, with 9489 trees and the highest relative abundance (RA) at 88.52%, among the dominant categories. It was followed by *Ginkgo biloba*, *Phoebe zhennan*, and *Ficus virens* var. *sublanceolata*, with 211 (1.97%), 169 (1.58%), and 154 (1.44%) trees, respectively. The above four top-ranking species contributed over 90% of the number of venerable trees in Sichuan. Trailing behind were 19 common species, each with >10 trees and an aggregate RA of approximately 4.44%; the number of trees of *Pistacia chinensis*, *Keteleeria davidiana*, *Platycladus orientalis*, *Cinnamomum camphora*, *Taxus wallichiana* var. *chinensis* was relatively higher in this group. The remaining 100 species had less than ten trees each, and their RA fell below the average of 0.02% per species. The long tail in the tree frequency data indicated the notable presence of rare (49) and solitary (51) species.

The top four species took up 92.10% of the relative dominance (RD), whereas the other 119 species contributed merely 7.90% (Table S3). The top six species had importance values (IV) above 1.0, with *C. funebris* dominating by a wide margin (172.49). The ensuing 28 species had IV between 0.1 and 1.0, while the remaining 89 species had IV below 0.1.

The above results indicated that *C. funebris* occupies a commanding position among venerable trees in Sichuan, reflecting the species’ long lifespan and environmental stress tolerance. However, the presence of a broad range of other common, rare and solitary species has enriched the diversity of the venerable stock, denoting that local people had attempted in the past to select well beyond the predominant species.

### 2.2. Elevation Distribution Patterns

**Figure 1:**
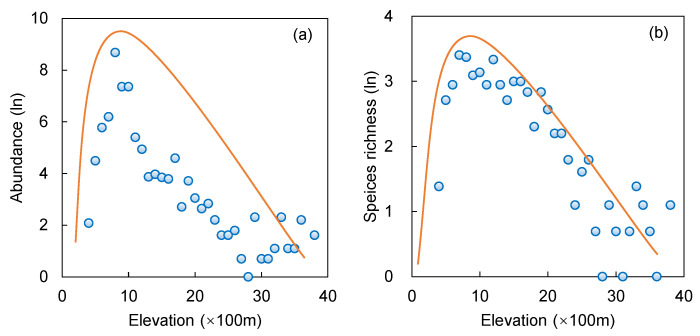
Vertical distribution patterns of the venerable trees in Sichuan in relation to: (**a**) tree abundance, and (**b**) species richness. The scatter points were fitted using a linear function.

The elevation distribution by tree abundance and species richness of venerable trees showed a unimodal pattern that skewed decidedly toward the low elevation end (Figure 1). The curves indicated a rapid increase in tree number within a narrow range at low elevation to reach a sharp peak, followed by a rather fast decrease thereafter, toward the high elevation end (Figure 1a). The maximum tree abundance (log value 8.67) occurred at 600–700 m, dominated by the presence of *C. funebris*. Meanwhile, the elevation steps of 600–700 m, 700–800 m and 800–900 m had more than 1500 trees each, totaling 8976 trees (83.73%) of the entire venerable tree population. In addition, no venerable trees were found in the 2600–2700 m, 3600–3700 m and 3700–3900 m zone. Therefore, the 600–900 m elevation bracket had the highest concentration of venerable trees.

The elevation distribution of species richness for venerable trees displayed a similar pattern to tree abundance, but with a smaller kurtosis (Figure 1b). The maximum value of species richness (log value 3.40 or 30 tree species) occurred at 500–600 m. It was followed by 600–700 m, 1000–1100 m, 800–900 m, 700–800 m, 1300–1400 m and 1400–1500 m, with species numbers 29, 28, 23, 22, 20 and 20, respectively. The number of species in other elevation steps was less than 20 each. Compared with the spread of high tree abundance, high species richness spread over a wider elevation span and reached a higher altitude.

### 2.3. Key Bioclimatic Factors

**Figure 2:**
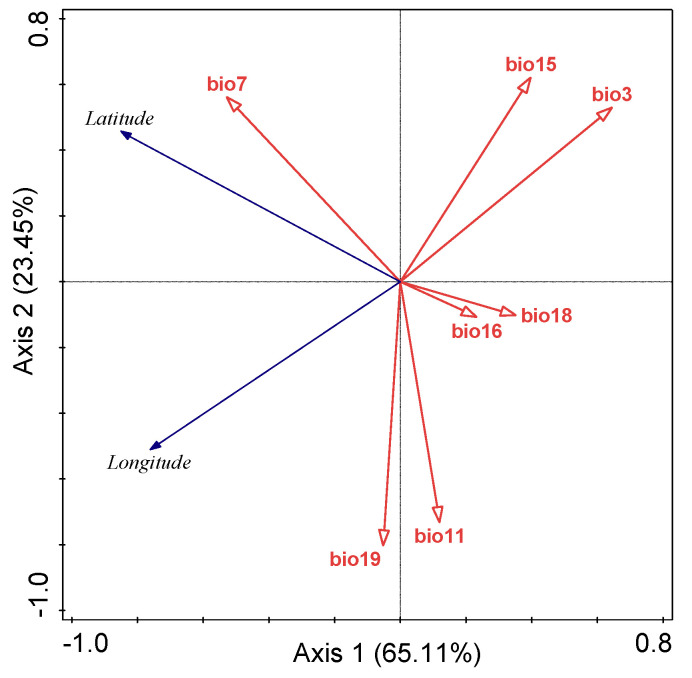
Ordination of geographical distribution and bioclimatic factors of venerable trees in Sichuan using redundancy analysis (RDA). See Table S1 for the meaning of bioclimatic factors. The solid arrowhead indicates the longitude and latitude as the dependent variable, and the hollow arrowhead indicates the bioclimatic factor as the independent variable. The included angle indicates the correlation (acute angle for positive correlation, obtuse angle for negative correlation, and right angle for no correlation).

The seven bioclimatic factors and 111 screened distribution coordinates of venerable trees in Sichuan were analyzed by RDA (Figure 2). Bio3, bio7, bio16, and bio18 played a major role in axis 1, with an aggregate contribution of 65.11%. Bio11, bio15 and bio19 played a major role in axis 2, with a contribution of 23.45% (Table 1). The cumulative contribution of the two axes was 88.56%, denoting an excellent explanation of the inter-variable relationships. The longitude was positively correlated with bio19 and bio11, and negatively correlated with bio15 and bio3. The latitude was positively correlated with bio7 and negatively correlated with bio16 and bio18.

The Monte-Carlo test evaluated the importance ranking of the seven bioclimatic factors (Table 1). All six bioclimatic factors had extremely significant effects (*p* < 0.01) on the distribution of venerable trees in Sichuan, with the exception of bio18. The bioclimatic factors were ranked in a descending sequence by their contribution to venerable-tree distribution: bio7 (38.3%) > bio3 (37.8%) > bio19 (10.4%) > bio15 (7.6%) > bio16 (3.0%) > bio11 (2.5%) > bio18 (0.4%). The results suggested that bio7, bio3 and bio19 played pivotal roles in the geographical distribution of venerable trees.

### 2.4. Current and Future Distribution Patterns

**Figure 3:**
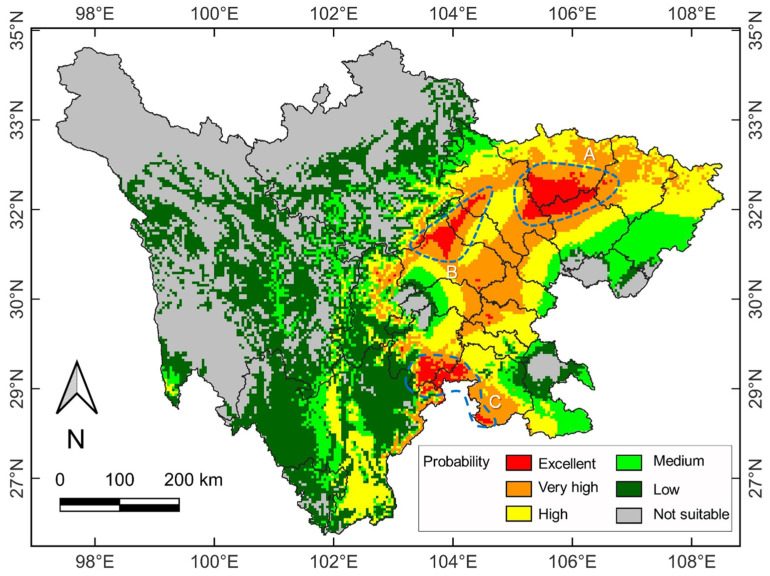
Distribution pattern of six categories of habitat suitability of venerable trees in Sichuan based on the current climate scenario. The results were obtained from the model predictions of BIOCLIM using DIVA-GIS. Different colors, from grey to red, denote the gradation from not suitable to excellent habitat suitability, and hence the probability of tree occurrence. The A, B and C annotations indicate the three largest patches of excellent suitability.

**Figure 4:**
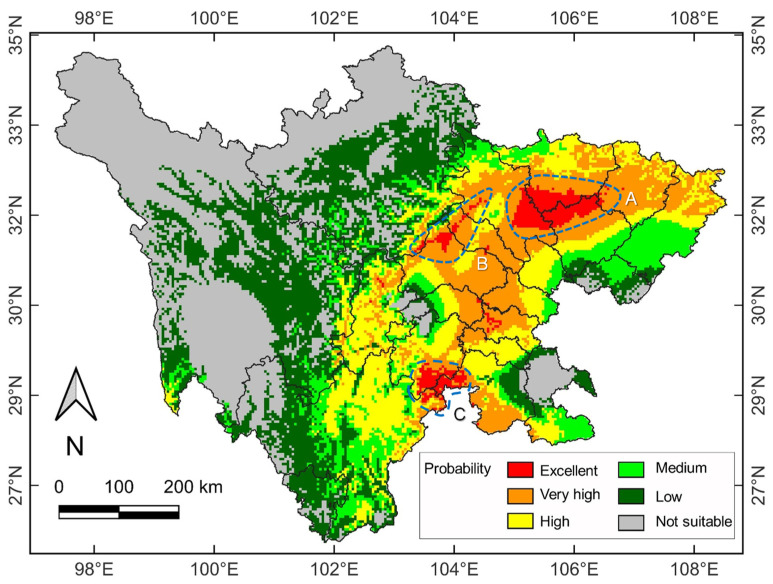
The potential biogeographical range of venerable trees in Sichuan under a future climate change scenario (double CO_2_ concentration according to the CCM3 model). Habitat suitability is classified into six categories denoted by different colors. The A, B and C annotations indicate the locations with a concentration of excellent habitat suitability.

Based on BIOCLIM, coupled with DIVA-GIS, 111 distribution points and seven bioclimatic variables were used to predict the suitable habitats of venerable trees in Sichuan under the current and future climate change scenarios (Figure 3 and Figure 4). The model’s forecast accuracy for the current climate scenario was judged to be ‘excellent’ (AUC, 0.911 ± 0.015). The findings confirmed that the chosen factors could accurately simulate the current distribution patterns. The excellent suitability habitat (the presence probability was 25–35%; shown in red in Figure 3) covered approximately 1.26 × 10^4^ km^2^ (Table S4). It was mainly concentrated in Sichuan’s eastern region, with the three large patches of excellent habitat marked A, B and C in Figure 3. They included Guangyuan, Nanchong, Mianyang, Deyang, and Chengdu, accompanied by small patches in Yibin and Leshan. The very high suitability habitat (10–20%, orange in Figure 3) surrounded the excellent one, covering an area of approximately 5.74 × 10^4^ km^2^. The high suitability habitat (the 5–10%, yellow in Figure 3) took up approximately 7.44 × 10^4^ km^2^. The combined area of the above three habitat categories amounted to 14.44 × 10^4^ km^2^, accounting for 29.63% of Sichuan’s total area. Therefore, not many suitable habitats for venerable trees were available under the current climate scenario. In addition, the suitability habitat of medium (2.5–5.0%, light green), low (0–2.5%, dark green) and not suitable (0%, gray) occupied 5.25 × 10^4^ km^2^, 13.08 × 104 km^2^ and 15.83 × 10^4^ km^2^, respectively. Overall, the suitability probability of venerable trees in eastern Sichuan was significantly better than that in western Sichuan under the current climate scenario, which should serve as a reference for the cultivation of potential trees to sustain the population.

The double CO_2_ concentration state in the 2100s was applied for the future climate change scenario. Compared with the current climate scenario, the suitability habitat pattern was somewhat modified (Figure 4). Firstly, the excellent habitat area was only marginally increased by 0.15% (12.58 × 10^4^ km^2^). However, the eastern parts of patches B and C (Figure 3) shrank slightly. In contrast, patch A was somewhat enlarged (Figure 4). Secondly, the very high and high suitability habitats increased to 6.79 × 10^4^ km^2^ (by 18.22%) and 7.82 × 10^4^ km^2^ (by 4.95%), respectively (Table S4). Meanwhile, the medium, low and not suitable suitability habitats decreased to 5.18 × 10^4^ km^2^ (by −1.13%), 11.76 × 10^4^ km^2^ (by −10.04%) and 15.79 × 10^4^ km^2^ (by −0.28%), respectively. Therefore, under the future warming scenario, the suitable habitat area for Sichuan’s venerable trees has been predicted to increase.

## 3. Discussion

With a long settlement history and inherently rich species diversity, Sichuan has preserved many venerable trees, denoting a valuable cultural and natural heritage [30]. The remnant natural forest fragments, often accommodating the venerable trees, play a role in preserving the outstanding natural emblems and regional ethnic culture and customs [22]. Such living relics are sometimes conceived as ecological barriers to human settlements and resource-tapping activities. Their conservation, more by default than by design, echoes the time-honored conception of striking a balance between protecting nature and satisfying human needs.

### 3.1. Characteristics of Venerable Trees Resources in Sichuan

Despite natural and human stresses, the venerable trees have survived through decades to centuries, owing to certain special biological and ecological characteristics [31]. These characteristics are closely related to their geographical location and regional culture [7,32]. However, in an area with an innately high biodiversity, it is rather perplexing to find such a substantial representation of *C. funebris* venerable trees in Sichuan (Table S3).

Firstly, *C. funebris* has a longer biological lifespan than other tree species [33]. Its wide ecological amplitude allows growth and distribution in a broad range in China [34]. This species prefers a warm and humid climate, with flexible adaptability to different soils, ranging from neutral to slightly acidic and calcareous [35]. With a broad environmental tolerance, it has been adopted as the main afforestation species in the southwestern mountains of China. It has been extensively planted on steep slopes with skeletal and infertile soil where vegetation restoration was difficult [36].

Secondly, *C. funebris* has been closely related to cultural traditions in western and eastern cultures since historical times. In the Roman period, coffins were often made of cypress wood (species in the genus *Cupressus*). Ancient Greeks and Romans would place cypress branches into coffins to bestow blessings of peace and happiness on the deceased [37]. Similarly, cypress trees have been planted in China’s graveyards since ancient times to convey the yearning for “immortality” to the departed. Since the Han Dynasty (206 BCE–220 CE), cypress trees have been commonly planted near graves during funerals [38,39]. Cypress trees have become a symbolic remembrance plant in China’s cemeteries.

Another explanation for the considerable abundance of *C. funebris* in Sichuan’s pool of venerable trees was its common planting as street trees in the past [40]. China has a long history of street-tree planting [41]. Thousands of *C. funebris* were cultivated along the edges of ancient post roads in Jiange County in the Ming Dynasty (1368–1644 CE). These roadside trees with a long lineage represent Sichuan’s largest and most well-protected batch of venerable trees [42]. In the past, the *C. funebris* street trees served multiple functions of living road signage, milestones, road surface protection, soil and water conservation, and wood supply [43]. Due to its well-known longevity and good performance in disturbed sites, the use of *C. funebris* as roadside trees could reduce the resources for replacement and maintenance and provide long-term landscape stability. Given these inherent qualities and performance track records, this species has continued to be widely planted in Sichuan in modern times.

In addition, *C. funebris* offers some important practical uses, including timber, traditional medicine, and aromatic essential oil. A long history of its cultivation and care, over two millennia, has provided a continuous pool of meritorious trees to join the privileged venerable assemblage. The varied direct and valuable functions have jointly contributed to its widespread cultivation and protection through the ages, contributing significantly to Sichuan’s venerable tree assets.

In addition to *C. funebris*, *Ginkgo biloba*, *Ficus virens* var. *sublanceolata*, and *Phoebe zhennan* were also found with fewer individuals, albeit significantly less than *C. funebris*. These species also interface intimately with human companions by playing important resource, amenity and cultural roles. For example, the ginkgo tree, regarded as a living fossil, was a relic species from the Quaternary Glaciation unique to China. The Chinese people have been fascinated by the ginkgo tree for several millennia. Deep respect has been elevated to the cultural phenomenon of worship [44]. Ginkgo trees have been traditionally cultivated and protected in the gardens of Buddhist temples. The steadfastly observed practice has boosted the interpretation of the ginkgo spirit and culture [45]. *P. zhennan* has a long history of cultivation in Sichuan and has been the exclusive prized timber for royalty since historical times [46]. Thus, these species have left their legacies in Sichuan’s pool of venerable trees.

### 3.2. Elevation Effect on Distribution Patterns

This study demonstrated that the distribution patterns of tree abundance and species richness tended to follow the mid-peak type (Figure 1). The tree abundance and species richness showed a unimodal curve, increasing with elevation, reaching the maximum value at low-middle elevation, then decreased afterward (Figure 1). However, the distribution was skewed toward the low-elevation end [47], indicating higher tree abundance and species richness at low-middle elevation, and lower at middle-high elevation.

At low elevations, intensive human activities were not conducive to Sichuan venerable-tree survival. Human impacts decreased with elevation, providing less stressful or better habitats for venerable trees. Meanwhile, the presence of the settlements could facilitate species dispersal [48], helping some tree species to disperse across the elevation gradient. Since ancient times, Sichuan has had a large population. The plains could not meet the agricultural needs of the sizeable population. People have gradually developed high-elevation areas to expand their farming activities, leading to mountain settlements [49]. The migrants provided opportunities to introduce and spread trees with economic and cultural utilities into the elevated lands.

With further elevation rise, human disturbances were reduced, and, correspondingly, the environmental role was enhanced to exert more influence on plant growth, which would be regulated by inherent physiological traits [50]. With few species suitable for the stressful high-altitude environment, tree abundance and species richness dropped after reaching a certain threshold altitude (Figure 1). Overall, elevation was one of the most direct factors limiting the distribution of venerable trees in Sichuan. Western Sichuan (Aba and Ganzi) is a plateau, and southeastern extension of the Tibetan Plateau and the Hengduan Mountains, with elevations up to 4000–4500 m (Figure S1) [51]. The cold and dry climate in western Sichuan limited the growth of venerable trees. Therefore, the high elevation explained the scarcity of venerable trees.

### 3.3. Current Potential Suitability Habitats and Key Environmental Variables

The current distribution pattern of venerable trees in Sichuan indicated that the eastern region was better endowed than the western, mainly explained by topographical differences. Sichuan is located in the transition zone between the Tibetan Plateau and the middle and lower reaches of the Yangtze River catchment. The geography of contrasting landforms and atmospheric circulation brought notable ecological divergence between the eastern and western regions [52]. The vegetation distribution displays significant horizontal and vertical zonal variations [51]. Eastern Sichuan falls in the humid subtropical climate zone, nurturing a wetter evergreen broad-leaved forest. The western region, with high elevation, undulating terrain, and cold climate is dominated by vegetation with a lower species diversity and simpler biomass structure. They include cold-tolerant subalpine coniferous forests, alpine oak forests, and alpine scrub meadow vegetation [53]. 

Our results show that the top two variables were closely related to temperature (Table 1, Figure 2). Their combined contribution value exceeded 75%, indicating the major role of temperature in determining venerable-tree distribution in Sichuan. Isothermality (bio3) and the temperature annual range (bio7) were pivotal in regulating the potential biogeographical range of venerable trees under the current climate. In contrast, factors associated more with moisture were relatively less important.

Plant distribution is contingent upon a package of factors, and each species tends to be determined by a key factor [54,55]. The climate is considered the principal factor driving the large-scale pattern of species distribution [56,57]. Both bio3 and bio7 reflected the temperature difference [58]. They were associated with three temperature-related factors, namely bio2 (mean diurnal range), bio5 (maximum temperature of the warmest month) and bio6 (minimum temperature of the coldest month). In fact, variations in temperature differences can directly or indirectly affect the uneven distribution of precipitation, which in turn can influence plant growth [59].

The distribution pattern of venerable trees was influenced by bio3 and bio7, particularly in eastern Sichuan, driven by temperature differences with an effect on plant growth and organic matter accumulation [60]. The distribution of Sichuan’s venerable trees was skewed pronouncedly toward higher longitudes (eastward), with higher mean temperatures in the warmest season fostering plant growth [61]. The venerable-tree distribution pattern also demonstrated a latitudinal gradient (Figure 2). This trend might be related to the temperature effect on seed germination, photosynthesis, respiration, and membrane stability [62].

### 3.4. Spatial Distribution under Future Climate Change Scenario

The species distribution pattern is an expression of adaptation to environmental changes. However, the intensification of the global warming process in the last century has impeded the species evolution rate, causing it to fall behind the pace of the temperature increase [63]. Such an out-of-phase change will substantially reduce the suitable habitats of many species in the coming years. This undesirable response is particularly evident for rare and endangered species that are sensitive to temperature [64]. Therefore, the impact of climate change on species distribution patterns should receive more attention.

Compared to the current suitability areas, major range changes in Sichuan’s venerable trees were forecasted under the future climate scenario (Figure 3 and Figure 4). The suitability habitats will increase in the very high and high suitability categories, coupled with a small increase in the excellent one (Table S4). Meanwhile, the medium, low, and not suitable habitat categories will decline. Overall, the favorable areas will experience range expansion, primarily contiguous to the current range patches, whereas the unfavorable will face contraction. This prediction signifies that climate warming will usher an overall positive effect on subtropical warmth-adapted trees.

Global warming can drive plant distribution to higher elevations and latitudes [65,66]. In the Pacific Northwest of North America, it has been predicted that Huckleberry (*Vaccinium membranaceum*) will expand into higher altitudes (>3050 m) by the end of the 21st century [67]. In Egypt, the suitable habitat of *Rosa arabica* may shift upward to the 1500–2000 m zone [68]. This study found that the habitat patch B located in the plains (Figure 3) was significantly reduced under climate warming (Figure 4). There were signs of migration from the plains to high elevations in Sichuan [69,70]. Although the area of very high and high suitability habitats may increase, the shrinkage or disappearance of excellent habitats in the plains indicate that climate warming could jeopardize the survival of venerable trees at low elevations.

## 4. Materials and Methods

### 4.1. Study Area

The study covered Sichuan, the second largest of China’s 23 provinces, with a land area of 4.85 × 10^5^ km^2^. It is located in the upper Yangtze River (Chang Jiang) catchment in the southwestern part of China (26°03′–34°19′ N, 97°21′–110°12′ E) (Figure S1). The relief of Sichuan’s eastern region contrasts sharply with the western. The extensive Sichuan Basin and its peripheral highlands dominate the eastern region, with the landform sloping toward the basin from all directions. The rugged terrain of the western region includes a plateau in the north and mountains in the south. Its north part is an extension of the Tibetan Plateau with highlands > 3700 m and higher mountain ranges.

The surrounding mountains sheltered the eastern region from cold polar air masses. The climate is milder than expected for its latitude and altitude, with more than 300 frost-free days, annually (less than 100 days in the western region). The mean annual temperature is 16–18 °C in the eastern region and is predominantly below 15 °C in the western region. The mean summer temperature in the eastern and western regions is 20–28 °C and 5–8 °C, and the mean winter temperature is 0.8–4.3 °C and <−10 °C, respectively. The eastern region’s annual precipitation measures about 1000 mm. Precipitation is lower in the western than in the eastern region by about 500–900 mm.

### 4.2. Collecting Venerable Tree Data

**Figure 5:**
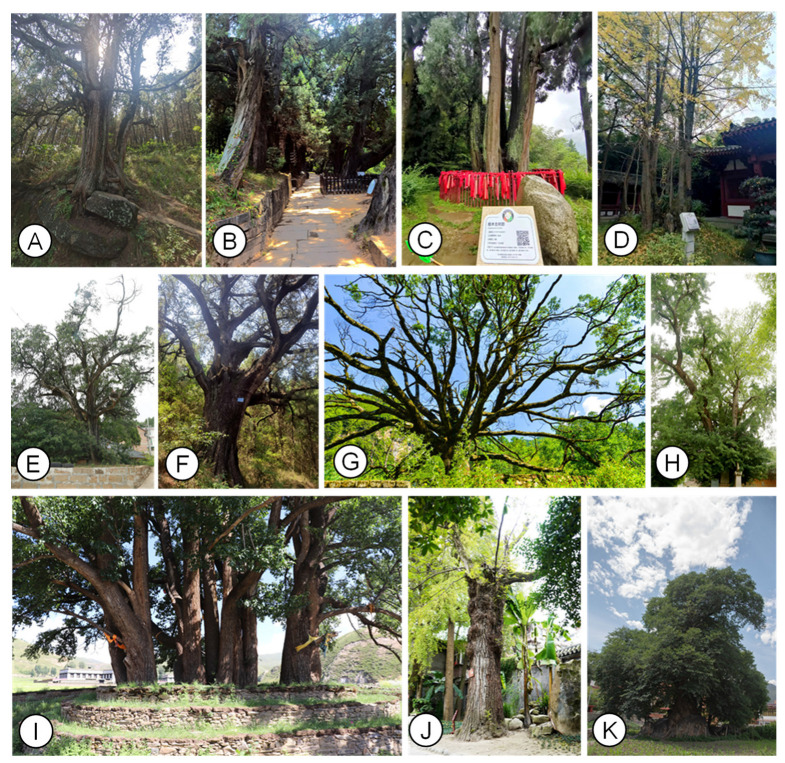
Photographs of main venerable trees and their habitats in Sichuan Province. Photo (**A**–**C**) and (**E**,**F**) denote *Cupressus funebris* (Cupressaceae); (**D**) and (**H**) *Ginkgo biloba* (Ginkgoaceae); (**G**) *Machilus nanmu* (Lauraceae); (**I**) *Cinnamomum camphora* (Lauraceae); (**J**) *Pterocarya stenoptera* (Juglandaceae); and (**K**) *Morus australis* (Moraceae).

This study is based on the “Announcement on the List of Old and Valuable Trees in Sichuan Province” (hereinafter referred to as “the announcement”), issued by the Sichuan Province Afforestation Committee [71]. In China, venerable trees are divided into three categories according to age: (1) >500 years; (2) 300–499 years and (3) 100–299 years [72]. However, determining the true age of a venerable tree is extremely difficult and complex [73]. Thus, we chose venerable trees thought to be older than 500 years in the announcement as study objects to ensure that only relatively old trees were incorporated into our study (Figure 5). The announcement includes essential data such as serial number, geographical location, elevation, age, height, diameter at breast height, and crown size.

To assist in tree sampling and calculating spatial distribution, the province was divided into 2.5′ × 2.5′ grids [74]. To reduce spatial autocorrelation [75], only one tree closest to the grid centroid was selected. After screening the data of 10,720 venerable trees of the announcement, 111 locations were selected to assess and predict the distribution pattern. 

### 4.3. Selecting Environmental Variables

Many studies employ 19 bioclimatic variables to simulate species distribution [75,76]. In this study, the current bioclimatic data to simulate suitable growth areas were extracted by DIVA-GIS 7.05 (WorldClim v.2.1 climate data for 1970–2000, http://www.worldclim.org/, accessed on 17 May 2020) [77]. They included the annual average temperature (bio1), annual precipitation (bio12), extreme high temperature (bio5), extreme low temperature (bio6), hottest season precipitation (bio18), coldest season precipitation (bio19), etc. (Table S1). Derived from monthly temperature and rainfall records, these bioclimatic factors represent annual trends, seasonality, and extreme or limiting environmental factors that are biologically meaningful [78]. The future climate data were tapped from the National Center for Atmospheric Research Community Climate Model, CCM3, which simulated a 2100s climate scenario with a double CO_2_ concentration [79].

The Pearson’s correlation coefficient (*r* value) was used to screen variables for multicollinearity, setting r at 0.80 as a threshold to eliminate correlated variables [80]. Variables that demonstrated multicollinearity with the largest number of variables were removed one at a time until none of the remaining variables had an *r* > 0.80 (Table S2).

### 4.4. Establishing the Species Distribution Model

The suitable areas for venerable trees in Sichuan were predicted in DIVA-GIS, using BIOCLIM [28,77]. We used 25% of the randomly selected points as test data to validate the models and the remaining 75% to train the models. This procedure was repeated ten times, and the mean AUC (area under the curve) values were used to determine model accuracy. The predicted distribution patterns generated by the BIOCLIM were imported into QGIS 3.20 software for visualization [81].

According to the default defined by DIVA-GIS, the predicted suitability areas of venerable trees should be divided into six grades, denoting a gradation of potential habitat suitability: excellent (20–42%), very high (10–20%), high (5–10%), medium (2.5–5.0%), low (0–2.5%), and not suitable (0%). The minimum geometric boundaries of the predicted spatial extent of the suitability grades were processed using QGIS, and the area of each grade was calculated (using the WGS84 coordinate system as the standard). The AUC, a quantitative indicator of model precision and strength, was used to evaluate its performance. A score of 0.5 indicates that the model performed poorly, 0.5–0.7 was reasonable, 0.7–0.9 was good, and >0.9 was excellent [55].

### 4.5. Statistical Analysis

The relative abundance (RA) and relative dominance (RD) of the trees were employed to derive the species importance value (IV) [82]. IV = RA + RD, where RA = number of trees in a species/total number of trees in the study area, and RD = basal area at breast height in a species/total basal area in the study area [14].

In this study, redundancy analysis (RDA) was performed in CANOCO 4.5 [83], using bioclimatic variables: latitude and longitude. The Monte-Carlo test was applied to quantify the contribution of each bioclimatic variable to the spatial distribution of venerable trees. It was used to determine the dominant bioclimatic variables affecting Sichuan’s distribution of venerable trees.

The elevation data of venerable trees were summarized and classified by 100 m steps. Tree abundance and species richness in each vertical step were summed up separately. Two scatter diagrams of the vertical distribution of venerable trees were plotted using elevation as the horizontal axis and tree abundance and species richness as the vertical axis.

## 5. Conclusions

Our work is the first study to evaluate the composition and spatial patterns of venerable trees in China’s Sichuan Province at the macro scale and to simulate their potential distribution under a climate change scenario. The computed importance values indicated an overwhelming domination by *C. funebris*, followed by *G. biloba*, *F. virens* var. *sublanceolata* and *P. zhennan*. The low-medium elevation at 600–1500 m was a relatively more favorable habitat for venerable trees in Sichuan. The bioclimatic factors indicated that isothermality (bio3) and temperature annual range (bio7) exerted major influences. 

The predicted current distribution matched its actual distribution, mainly occurring in eastern Sichuan. Under the future climate scenario of doubled CO_2_ concentration, the model predicted the expansion of suitable habitats for Sichuan’s venerable trees. However, the local reduction in excellent habitats, mainly situated in the low-altitude plains, implied some risk to venerable-tree survival due to climate warming.

The findings can inform the formulation of conservation guidelines for venerable trees in Sichuan. They provide a reference to assess key cultivation areas for planting potential venerable trees and to develop strategies for preserving existing resources, particularly in the most favorable sites. Locations with high-grade suitability habitats predicted to have no range change in the face of climate change can be identified as potential refugia. Where appropriate, such valuable sites could be considered for protected-area designation.

## Figures and Tables

**Table 1:** Monte-Carlo test results of geographical distribution and bioclimatic factors of venerable trees in Sichuan, ranked by their contributions.

Bioclimatic Factor	Explained Variation %	Contribution %	*F*	*p*
bio7	34.0	38.3	113	0.002
bio3	33.5	37.8	54.9	0.002
bio19	9.2	10.4	42.0	0.002
bio15	6.7	7.6	43.0	0.002
bio16	2.6	3.0	19.8	0.002
bio11	2.2	2.5	19.5	0.002
bio18	0.3	0.4	3.1	0.066

## Data Availability

The data presented in this study are available on request from the corresponding author.

## Supplementary Materials

The following supporting information can be downloaded at: https://www.mdpi.com/article/10.3390/plants11243581/s1, Figure S1: The locations of occurrence records (shown by white dots) of venerable trees in relation to elevation in Sichuan Province, China; Table S1: Nineteen environmental variables that may predict the geographical distribution of venerable trees in Sichuan; Table S2: Pearson correlation coefficient matrix of seven chosen environmental variables to predict the geographical distribution of venerable trees in Sichuan.; Table S3: Species composition and importance values of venerable trees in Sichuan, ranked by importance values (IV); Table S4: Predicted suitable habitats for venerable trees in Sichuan under current and future climate scenarios.
