# Supplementary material for: Environmental Factors Driving the Spatial Distribution Pattern of Venerable Trees in Sichuan Province, China
Source: Plants (Basel). 2022 Dec 19;11(24):3581. doi: 10.3390/plants11243581 (PMC9780929; doi:10.3390/plants11243581)
Supplement: Supplementary file 1 [file plants-11-03581-s001.zip › plants-2081494-supplementary.pdf]

Supplementary materials:

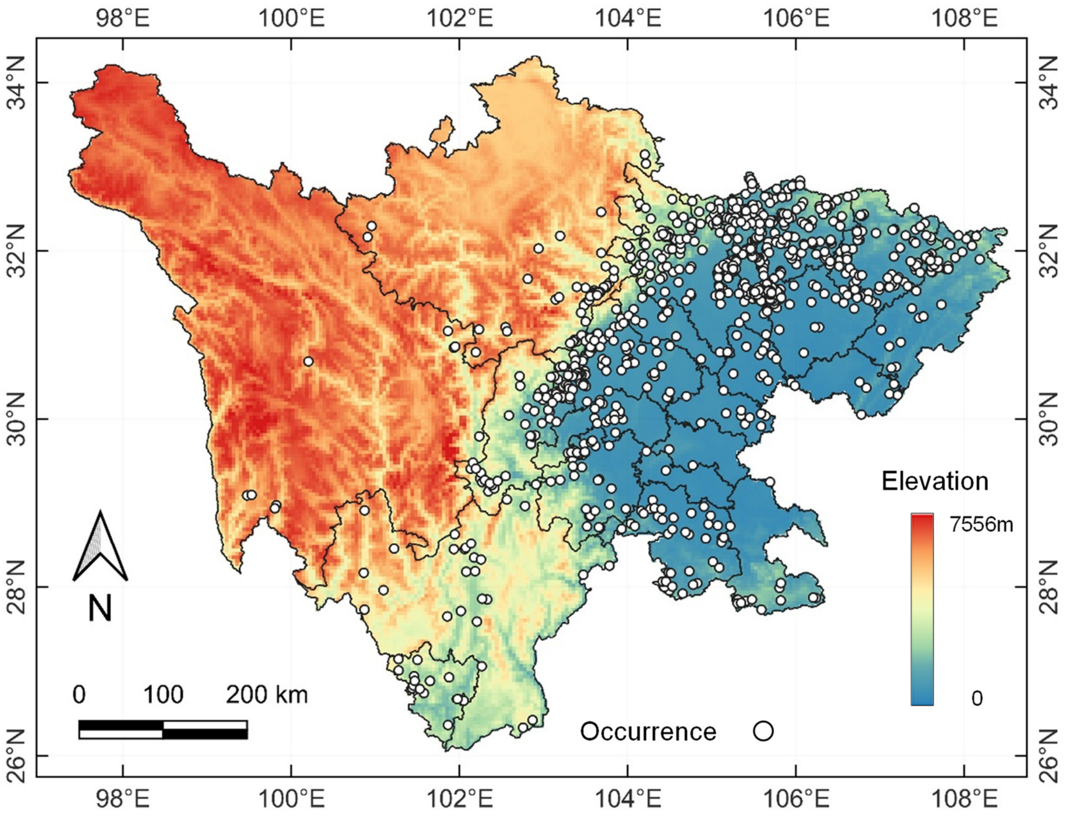

Figure S1. The locations of occurrence records (shown by white dots) of venerable trees in relation to elevation in Sichuan Province, China.

Table S1. Nineteen environmental variables that may predict the geographical distribution of venerable trees in Sichuan.

Bioclimatic variable	Code	Unit	Bioclimatic variable	Code	Unit
Annual mean temperature	Bio 1	℃	Mean temperature of coldest quarter	Bio 11	℃
Mean diurnal range	Bio 2	℃	Annual precipitation	Bio 12	mm
Isothermality (Bio2/Bio7) (×100)	Bio 3	Index	Precipitation of wettest month	Bio 13	mm
Temperature seasonality	Bio 4	Index	Precipitation of driest month	Bio 14	mm
Max temperature of warmest month	Bio 5	℃	Precipitation seasonality	Bio 15	Index
Min temperature of coldest month	Bio 6	℃	Precipitation of wettest quarter	Bio 16	mm
Temperature annual range	Bio 7	℃	Precipitation of driest quarter	Bio 17	mm
Mean temperature of wettest quarter	Bio 8	℃	Precipitation of warmest quarter	Bio 18	mm
Mean temperature of driest quarter	Bio 9	℃	Precipitation of coldest quarter	Bio 19	mm
Mean temperature of warmest quarter	Bio 10	℃			

Table S2. Pearson correlation coefficient matrix of seven chosen environmental variables to predict the geographical distribution of venerable trees in Sichuan.

Variable[a]	bio3	bio7	bio11	bio15	bio16	bio18	bio19
bio3	1.000						
bio7	-0.026	1.000					
bio11	-0.317**	-0.579**	1.000				
bio15	0.272**	-0.200**	0.118**	1.000			
bio16	-0.079**	-0.137**	0.154**	0.041	1.000		
bio18	-0.180**	-0.605**	0.425**	0.443**	0.105**	1.000	
bio19	-0.492**	-0.525**	0.440**	-0.381**	0.076**	0.577**	1.000

[a] The meanings of bioclimatic variables can be found in Table S1.
** Denotes that the difference is significant at the 0.01 level.

Table S3. Species composition and importance values of venerable trees in Sichuan, ranked by importance values (IV).

Scientific name	Family	DBH	Count	RD[a]	RA[a]	IV
Cupressus funebris	Cupressaceae	2,665,828	9489	83.972	88.517	172.489
Ginkgo biloba	Ginkgoaceae	98,418	211	3.100	1.968	5.068
Ficus virens var. *sublanceolata*	Moraceae	105,223	154	3.314	1.437	4.751
Phoebe zhennan	Lauraceae	54,531	169	1.718	1.576	3.294
Pistacia chinensis	Anacardiaceae	35,169	95	1.108	0.886	1.994
Keteleeria davidiana	Pinaceae	22,987	50	0.724	0.466	1.190
Platycladus orientalis	Cupressaceae	14,364	37	0.452	0.345	0.798
Cinnamomum camphora	Lauraceae	11,098	29	0.350	0.271	0.620
Taxus wallichiana var. *chinensis*	Taxaceae	10,332	30	0.325	0.280	0.605
Pterocarya stenoptera	Juglandaceae	11,542	22	0.364	0.205	0.569
Juglans regia	Juglandaceae	10,822	19	0.341	0.177	0.518
Gleditsia sinensis	Caesalpiniaceae	6991	24	0.220	0.224	0.444
Melia azedarach	Meliaceae	6017	23	0.190	0.215	0.404
Ormosia hosiei	Papilionaceae	5405	23	0.170	0.215	0.385
Styphnolobium japonicum	Papilionaceae	7142	17	0.225	0.159	0.384
Cyclobalanopsis glauca	Fagaceae	6208	18	0.196	0.168	0.363
Liquidambar formosana	Hamamelidaceae	6113	16	0.193	0.149	0.342
Ligustrum lucidum	Oleaceae	4468	12	0.141	0.112	0.253
Podocarpus macrophyllus	Podocarpaceae	3591	14	0.113	0.131	0.244
Xylosma congesta	Flacourtiaceae	3412	14	0.107	0.131	0.238
Lagerstroemia indica	Lythraceae	2568	12	0.081	0.112	0.193
Litchi chinensis	Sapindaceae	2829	10	0.089	0.093	0.182
Emmenopterys henryi	Rubiaceae	3866	5	0.122	0.047	0.168
Osmanthus fragrans	Oleaceae	1855	11	0.058	0.103	0.161
Acer oblongum	Aceraceae	2309	9	0.073	0.084	0.157
Cycas revoluta	Cycadaceae	2372	8	0.075	0.075	0.149
Pinus massoniana	Pinaceae	1942	8	0.061	0.075	0.136
Pistacia weinmanniifolia	Anacardiaceae	2081	7	0.066	0.065	0.131
Keteleeria evelyniana	Pinaceae	2290	5	0.072	0.047	0.119
Choerospondias axillaris	Anacardiaceae	2495	4	0.079	0.037	0.116
Cunninghamia lanceolata	Taxodiaceae	1994	5	0.063	0.047	0.109
Machilus nanmu	Lauraceae	1570	6	0.049	0.056	0.105
Bombax ceiba	Bombacaceae	2196	3	0.069	0.028	0.097
Camellia sinensis	Theaceae	321	9	0.010	0.084	0.094
Juniperus chinensis	Cupressaceae	1463	5	0.046	0.047	0.093
Meliosma veitchiorum	Sabiaceae	2330	2	0.073	0.019	0.092
Cupressus chengiana	Cupressaceae	1568	4	0.049	0.037	0.087
Michelia wilsonii	Lauraceae	1557	4	0.049	0.037	0.086
Populus davidiana	Salicaceae	1547	4	0.049	0.037	0.086
Tilia tuan	Tiliaceae	2104	2	0.066	0.019	0.085
Quercus semecarpifolia	Fagaceae	1585	3	0.050	0.028	0.078
Quercus acutissima	Fagaceae	1282	4	0.040	0.037	0.078
Lindera megaphylla	Lauraceae	1387	3	0.044	0.028	0.072
Picea asperata	Pinaceae	1356	3	0.043	0.028	0.071
Pinus bungeana	Pinaceae	1020	4	0.032	0.037	0.069
Diospyros nigrocortex	Ebenaceae	1300	3	0.041	0.028	0.069
Populus tomentosa	Salicaceae	1350	2	0.043	0.019	0.061
Castanopsis platyacantha	Fagaceae	946	3	0.030	0.028	0.058

Abies fabri	Pinaceae	852	3	0.027	0.028	0.055
Tsuga dumosa	Pinaceae	1130	2	0.036	0.019	0.054
Tsuga chinensis	Pinaceae	818	3	0.026	0.028	0.054
Lagerstroemia excelsa	Lythraceae	767	3	0.024	0.028	0.052
Kalopanax septemlobus	Araliaceae	1002	2	0.032	0.019	0.050
Litsea coreana var. *lanuginosa*	Lauraceae	624	3	0.020	0.028	0.048
Ehretia dicksonii	Boraginaceae	885	2	0.028	0.019	0.047
Camellia gymnogyna	Theaceae	587	3	0.018	0.028	0.046
Pseudotsuga sinensis	Pinaceae	848	2	0.027	0.019	0.045
Ficus religiosa	Moraceae	535	3	0.017	0.028	0.045
Ziziphus jujuba var. *spinosa*	Rhamnaceae	816	2	0.026	0.019	0.044
Juniperus saltuaria	Cupressaceae	785	2	0.025	0.019	0.043
Celtis sinensis	Ulmaceae	681	2	0.021	0.019	0.040
Populus cathayana	Salicaceae	950	1	0.030	0.009	0.039
Cinnamomum longepaniculatum	Lauraceae	625	2	0.020	0.019	0.038
Acer pictum subsp. *mono*	Aceraceae	614	2	0.019	0.019	0.038
Quercus variabilis	Fagaceae	603	2	0.019	0.019	0.038
Cyclobalanopsis gracilis	Fagaceae	288	3	0.009	0.028	0.037
Vitex negundo	Verbenaceae	554	2	0.017	0.019	0.036
Radermachera microcalyx	Bignoniaceae	541	2	0.017	0.019	0.036
Cupressus duclouxiana	Cupressaceae	540	2	0.017	0.019	0.036
Quercus spinosa	Fagaceae	512	2	0.016	0.019	0.035
Engelhardia roxburghiana	Juglandaceae	785	1	0.025	0.009	0.034
Ormosia yaanensis	Papilionaceae	785	1	0.025	0.009	0.034
Acer buergerianum	Aceraceae	487	2	0.015	0.019	0.034
Morus australis	Moraceae	760	1	0.024	0.009	0.033
Lindera aggregata	Lauraceae	442	2	0.014	0.019	0.033
Cercis chinensis	Caesalpiniaceae	439	2	0.014	0.019	0.032
Meliosma alba	Sabiaceae	637	1	0.020	0.009	0.029
Acer flabellatum	Aceraceae	584	1	0.018	0.009	0.028
Juniperus squamata	Cupressaceae	565	1	0.018	0.009	0.027
Populus alba	Salicaceae	540	1	0.017	0.009	0.026
Bischofia polycarpa	Euphorbiaceae	502	1	0.016	0.009	0.025
Yulania liliiflora	Magnoliaceae	495	1	0.016	0.009	0.025
Ailanthus altissima	Simaroubaceae	480	1	0.015	0.009	0.024
Diospyros kaki	Ebenaceae	480	1	0.015	0.009	0.024
Sorbus pohuashanensis	Rosaceae	480	1	0.015	0.009	0.024
Photinia glomerata	Rosaceae	452	1	0.014	0.009	0.024
Laurocerasus zippeliana	Rosaceae	408	1	0.013	0.009	0.022
Lithocarpus confinis	Fagaceae	408	1	0.013	0.009	0.022
Aidia canthioides	Rubiaceae	404	1	0.013	0.009	0.022
Celtis biondii	Ulmaceae	399	1	0.013	0.009	0.022
Castanea mollissima	Fagaceae	393	1	0.012	0.009	0.022
Quercus acrodonta	Fagaceae	380	1	0.012	0.009	0.021
Albizia kalkora	Mimosaceae	377	1	0.012	0.009	0.021
Buxus sinica	Buxaceae	377	1	0.012	0.009	0.021
Dalbergia hupeana	Papilionaceae	360	1	0.011	0.009	0.021
Elaeocarpus japonicus	Elaeocarpaceae	330	1	0.010	0.009	0.020
Morus macroura	Moraceae	316	1	0.010	0.009	0.019
Rosa chinensis	Rosaceae	308	1	0.010	0.009	0.019
Beilschmiedia delicata	Lauraceae	304	1	0.010	0.009	0.019
Sloanea sinensis	Elaeocarpaceae	301	1	0.009	0.009	0.019
Yulania dawsoniana	Magnoliaceae	298	1	0.009	0.009	0.019

Pinus yunnanensis	Pinaceae	289	1	0.009	0.009	0.018
Pyrus betulifolia	Rosaceae	260	1	0.008	0.009	0.018
Quercus mongolica	Fagaceae	242	1	0.008	0.009	0.017
Crataegus pinnatifida	Rosaceae	229	1	0.007	0.009	0.017
Michelia martini	Magnoliaceae	226	1	0.007	0.009	0.016
Morus alba	Moraceae	220	1	0.007	0.009	0.016
Ulmus parvifolia	Ulmaceae	207	1	0.007	0.009	0.016
Wisteria sinensis	Papilionaceae	206	1	0.006	0.009	0.016
Quercus aquifolioides	Fagaceae	204	1	0.006	0.009	0.016
Ziziphus jujuba	Rhamnaceae	200	1	0.006	0.009	0.016
Idesia polycarpa var. *vestita*	Flacourtiaceae	165	1	0.005	0.009	0.015
Cinnamomum japonicum	Lauraceae	160	1	0.005	0.009	0.014
Cercidiphyllum japonicum	Cercidiphyllaceae	143	1	0.005	0.009	0.014
Castanea henryi	Fagaceae	138	1	0.004	0.009	0.014
Myrica rubra	Myricaceae	138	1	0.004	0.009	0.014
Camellia japonica	Theaceae	110	1	0.003	0.009	0.013
Diospyros oleifera	Ebenaceae	102	1	0.003	0.009	0.013
Armeniaca mume	Rosaceae	100	1	0.003	0.009	0.012
Amygdalus persica	Rosaceae	95	1	0.003	0.009	0.012
Morus mongolica	Moraceae	88	1	0.003	0.009	0.012
Diospyros armata	Ebenaceae	79	1	0.002	0.009	0.012
Koilodepas hainanense	Euphorbiaceae	65	1	0.002	0.009	0.011
Total		3,174,644	10,720	100.000	100.000	200.000

[a] RD denotes relative dominance, and RA relative abundance.

Table S4. Predicted suitable habitats for venerable trees in Sichuan under current and future climate scenarios.

**Suitability category**	**Predicted area (x 10^4 km^2)**		Area change (x 10^4 km^2)	Change ratio (%)
	**Current**	Future		
**Excellent**		12,579	18	0.15
Very high	57,398	67,856	10,458	18.22
High	74,495	78,184	3689	4.95
Medium	52,455	51,865	-590	-1.13
Low	130,750	117,618	-13,132	-10.04
Not suitable	158,342	157,899	-443	-0.28
